# A breast cancer meta-analysis of two expression measures of chromosomal instability reveals a relationship with younger age at diagnosis and high risk histopathological variables

**DOI:** 10.18632/oncotarget.298

**Published:** 2011-06-25

**Authors:** David Endesfelder, Nicholas McGranahan, Nicolai J. Birkbak, Zoltan Szallasi, Maik Kschischo, Trevor A. Graham, Charles Swanton

**Affiliations:** ^1^Cancer Research UK London Research Institute, London, WC2A 3LY, United Kingdom.; ^2^Centre for Mathematics and Physics in the Life Sciences and Experimental Biology (CoMPLEX), University College London, Physics Building, Gower Street, London, WC1E 6BT; ^3^Department of Cancer Biology, Dana-Farber Cancer Institute, Harvard Medical School, Boston, Massachusetts, USA.; ^4^Center for Biological Sequence Analysis, Technical University of Denmark, DK-2800 Lyngby, Denmark.; ^5^Children's Hospital Informatics Program at the Harvard-MIT Division of Health Sciences and Technology (CHIP@HST), Harvard Medical School, Boston, MA 02115, USA.; ^6^University of Applied Sciences, Südallee 2, 53424 Remagen, Germany.; ^7^Royal Marsden Hospital, Breast and Drug Development Units, Dept Medicine, Sutton, SM2 5PT, UK.

**Keywords:** breast cancer, age, chromosomal instability, histopathological parameters

## Abstract

Breast cancer in younger patients often presents with adverse histopathological features, including increased frequency of estrogen receptor negative and lymph node positive disease status. Chromosomal instability (CIN) is increasingly recognised as an important prognostic variable in solid tumours. In a breast cancer meta-analysis of 2423 patients we examine the relationship between clinicopathological parameters and two distinct chromosomal instability gene expression signatures in order to address whether younger age at diagnosis is associated with increased tumour genome instability. We find that CIN, assessed by the two independently derived CIN expression signatures, is significantly associated with increased tumour size, ER negative or HER2 positive disease, higher tumour grade and younger age at diagnosis in ER negative breast cancer. These data support the hypothesis that chromosomal instability may be a defining feature of breast cancer biology and clinical outcome.

## INTRODUCTION

Breast cancer in younger women has been shown to be associated with a worse prognosis than in older women [1-3]. Risk factors, including high tumour grade, large tumour size, positive lymph node, and Estrogen receptor (ER) negative status, have been shown to be more prevalent in younger breast cancer patients, leading some to suggest that breast cancer in younger women represents a distinct clinical entity[4].

Chromosomal instability has been widely documented to be associated with poorer prognosis in solid tumours [5] and CIN induced by MAD2 expression promotes rapid tumour relapse following withdrawal of an oncogenic stimulus in animal models [6]. However pre-clinical models have in some cases demonstrated a deleterious impact upon cancer cell survival associated with excessive chromosomal instability initiated by spindle assembly checkpoint inactivation [7] and we and others have shown that aneuploidy has a negative impact upon cell proliferation [8]. Indeed, animal models have also demonstrated that aneuploidy may confer a tumour suppressor effect in cancer prone mice[9]. Cahill and colleagues have suggested that whilst genetic instability may be advantageous under tumour-stromal selection pressures, a threshold may exist beyond which excessive instability becomes deleterious for cancer survival[10].

We reasoned that the association of CIN with poorer prognosis may mask more subtle associations and that extreme CIN predicts improved outcome in contrast to intermediate CIN that might be associated with poorer prognosis. We have previously demonstrated that there may be a non-monotonic relationship between CIN and clinical outcome in a retrospective analysis of breast cancer outcome. We have used a surrogate of CIN status, assessed by CIN70 expression signature, which we have shown acts as a robust surrogate of structural chromosomal complexity measured by CGH and numerical CIN assessed by DNA image cytometry[11]. In this study, we separated patients into quartiles of CIN70 expression and identified that patients with ER negative breast cancer with CIN extreme (4^th^ quartile of CIN70 expression) appear to have improved outcome relative to patients with tumours in the intermediate 3^rd^ quartile which were associated with the worst outcome. In summary, these data confirm the association of CIN with poorer outcome, but also suggest that extreme CIN, exceeding a certain threshold, is associated with improved prognosis.

Here we assess the relationship between CIN70 quartile, which we have previously demonstrated correlates with both numerical chromosomal instability and structural chromosomal complexity, and breast cancer histopathological parameters and age at diagnosis. Additionally, we determine the relationship of histopathological parameters with a breast cancer genomic instability signature derived by Habermann et al.[12]. We find evidence for a significant association of both chromosomal instability signatures with high risk histopathological features and importantly with younger age at diagnosis in ER negative patients in a meta-analysis of 2423 patients. These data are concordant with recent suggestions that breast cancer in younger women represents a distinct clinical entity with higher risk molecular features[4].

## RESULTS

### Comparison of CIN70 quartiles with the 12 gene genome instability signature

**Figure 1:**
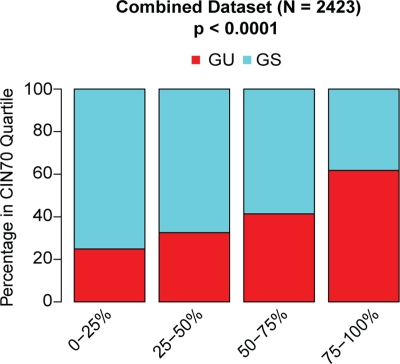
Association of CIN70 gene expression signature and the 12 gene genomic instability signature Proportions of genomically unstable (GU) and genomically stable (GS) patients, derived by the 12-gene genomic instability signature, with CIN70 score quartiles.

We used two different methods to assess chromosomal instability across large cohorts of patients with primary breast cancer for which tumour microarray gene expression data were available. Firstly, we used the CIN70 expression signature derived from a measure of total functional aneuploidy [13]. We have previously shown that this measure correlates with both numerical CIN and structural chromosomal complexity in breast cancer [11]. Secondly, we used a 12-gene genome instability signature defining genomically unstable (GU) breast cancers correlating gene expression data with chromosomal instability in breast cancer measured by DNA image cytometry, previously derived by Habermann and colleagues [12]. In this study, aneuploid genomically unstable breast cancers were defined as having the broadest distribution of DNA content. To compare the two signatures, we derived quartiles of the CIN70 expression scores, as previously described[11], in a meta-analysis of gene expression datasets deriving from 2423 patients with primary breast cancer (Table 1). We assessed the representation of genomically unstable (aGU) and genomically stable (GS) tumours based on the 12 gene signature within each CIN70 expression quartile (Figure 1). We found a significant trend of increasing proportions of genomically unstable tumours with increasing CIN70 quartiles (Figure 1, p < 0.0001), suggesting that both chromosomal instability expression signatures derived through independent methods, are highly correlated.

**Table 1:** Association of chromosomal instability signatures with clinical parameters. Association of CIN70 quartiles and genomic instability grouped by the 12 gene signature with age, grade, tumour size, lymph node status, ER status and HER2 status.

	**CIN70 0 – 25%**	**CIN70 25 – 50%**	**CIN70 50 – 75%**	**CIN70 75 – 100%**	**Habermann GS**	**Habermann GU**
**Age (N, %)**						
** Median Age (Range)**	55 (25–88)	55 (25–95)	55 (24–93)	52 (24–88)	55 (24 – 88)	54 (25 – 95)
***Age < 45***	70 (16)	71 (17)	89 (21)	107 (25)	187 (19)	150 (21)
***Age –***	45 369 (84)	341 (83)	335 (79)	328 (75)	801 (81)	572 (79)
***Unknown***	176	185	181	171	475	238
**Grade (N, %)**						
***1***	147 (39)	68 (19)	32 (9)	10 (3)	217 (25)	40 (7)
***2***	188 (50)	197 (55)	128 (37)	61 (16)	383 (44)	191 (33)
***3***	40 (11)	93 (26)	185 (54)	311 (81)	276 (31)	353 (60)
***Unknown***	240	239	260	224	587	376
**ER Status (N, %)**						
***ER Positive***	515 (89)	440 (79)	355 (74)	194 (34)	1156 (85)	348 (39)
***ER Negative***	64 (11)	115 (21)	197 (36)	372 (66)	204 (15)	544 (61)
***Unknown***	36	42	53	40	103	68
**HER2 Status**						
***HER2 Positive***	21 (4)	90 (17)	127 (24)	113 (21)	158 (12)	193 (26)
***HER2 Negative***	532 (96)	438 (83)	393 (76)	427 (79)	1129 (88)	661 (74)
***Unknown***	62	69	85	66	176	106
**Size (N, %)**						
***0 – 2 cm***	174 (60)	156 (57)	110 (43)	99 (35)	374 (53)	165 (42)
***> 2cm***	117 (40)	116 (43)	146 (57)	182 (65)	335 (47)	226 (57)
***Unknown***	324	325	349	325	754	569
**Lymph Node (N, %)**						
***Positive***	89 (24)	101 (29)	95 (30)	90 (26)	250 (28)	125 (26)
***Negative***	278 (76)	248 (71)	219 (70)	253 (74)	633 (72)	365 (74)
***Unknown***	248	248	291	263	580	470

### CIN status and histopathological parameters

**Figure 2:**
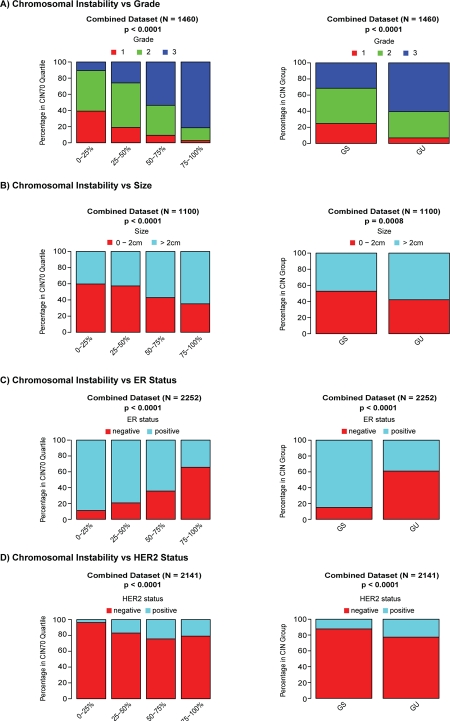
Association of genomic instability expression signatures with clinical parameters Association of CIN70 quartiles (left) and the 12-gene genomic instability signature (right) with tumour grade (Figure 2A), tumour size (Figure 2B), ER status (Figure 2C) and HER2 status (Figure 2D).

To assess the association of CIN70 expression scores with breast cancer histopathological variables we performed a meta-analysis of 13 primary breast cancer expression datasets (n=2423 patients). We confirmed that tumours of higher grade are enriched with increasing chromosomal instability scores assessed by the CIN70 signature (Figure 2A, p < 0.0001) [13]. Increasing CIN70 quartile correlated with higher risk breast cancer histopathological features including larger tumour size (Figure 2B, p < 0.0001) and the proportion of patients with ER negative (Figure 2C, p < 0.0001) or HER2 positive (Figure 2D, p < 0.0001) breast cancer. We detected no association of CIN70 quartiles with lymph node status (data not shown). In addition, we assessed the association of histopathological parameters with tumours grouped by genomically unstable (GU) or genomically stable (GS) status based on the independently derived 12 gene genomic instability expression signature [12]. Consistent with the results obtained with the CIN70 signature, we observed that tumours of higher grade (Figure 2A, p < 0.0001), larger size (Figure 2B, p = 0.0008), ER negative (Figure 2C, p < 0.0001) or HER2 positive (Figure 2D, p < 0.0001) status are enriched in the GU group. Similar to the CIN70 analysis, no association of lymph node status with GU was observed (data not shown).

### Assessment of CIN status with age of breast cancer diagnosis

**Figure 3:**
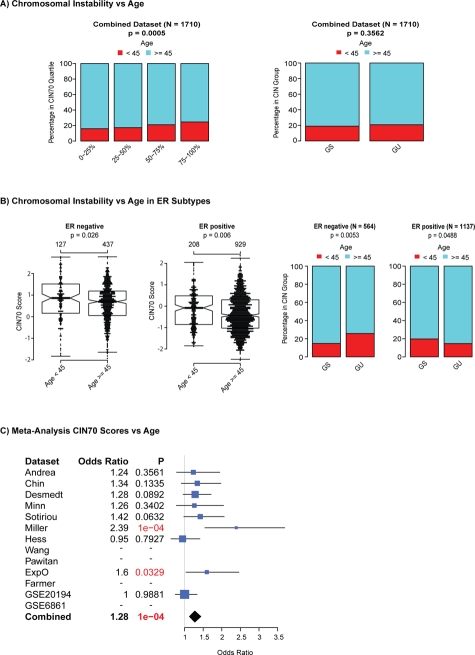
Association of genomic instability expression signatures with age at diagnosis Association of age with CIN70 quartiles (left) and the 12-gene genomic instability signature (right) for all patients. (Figure 3B) Association of CIN70 scores and the 12-gene genomic instability signature in ER negative and ER positive breast cancer. (Figure 3C): Forestplot showing the meta-analysis results of a logistic regression assessing the odds ratio for the younger patient group vs. the older patient group as CIN70 scores increase.

To address whether there is a relationship between chromosomal instability status and younger age of diagnosis we analysed 8 microarray datasets, for which age data were available, comprising 1710 patients and classified patients into two groups based on age < 45 years (N = 337) and ≥45 years (N = 1373), a standard threshold used to separate breast cancer patients. Firstly, we tested for differences in CIN70 scores between younger and older patients for the combined dataset and found that CIN70 scores and CIN70 expression quartiles were significantly higher in tumours from younger patients (Figure 3A, p < 0.00005). In contrast, we observed no significant association of aGU tumours in younger patients in the combined dataset (Figure 3A, p = 0.3562). We examined ER negative and ER positive breast cancers separately and found a significant association of higher CIN70 expression with younger age in both ER positive and ER negative breast cancer (Figure 3B, ER negative p = 0.026 ER positive p = 0.006 suggesting that the differences in CIN70 scores are independent of ER status. A significant association of younger age with aGU tumours was detected in ER negative breast cancer (Figure 3B, p = 0.0053) but not in ER positive breast cancer (Figure 3B). Finally, we used logistic regression models to test for a significantly higher probability for patients being in the younger patient group as CIN70 scores increase. We found significant differences in 2 out of 9 datasets and a trend for patients with higher CIN70 scores being in the younger patient group in 5 out of 9 datasets (Figure 3C). When all datasets were combined, we found a highly significant association of younger age at diagnosis with increased CIN70 scores (p < 0.0001). In summary, we have identified evidence for the enrichment of CIN, using two independently derived CIN signatures, in tumours from younger patients with ER negative breast cancer.

## DISCUSSION

Clinical and histopathological analyses in breast cancer have suggested that cancer in younger women appears to be associated with high-risk histopathological features and worse clinical outcome. Chromosomal instability represents one recognised prognostic feature in solid tumours. Given the association of a surrogate measure of CIN, the CIN70 expression signature, with both numerical CIN and structural chromosomal complexity [11], we were able to determine that tumours from younger patients with breast cancer were more likely to be characterised by high CIN70 expression levels in both ER positive and ER negative breast cancer. We also demonstrate, using a second independently derived measure of CIN, the 12 gene genome instability score, that GU/CIN tumours are significantly enriched in ER negative breast cancers from younger patients relative to patients with ER negative breast cancer older than 45 years at diagnosis. However, we observed divergent results for ER positive breast cancers, where tumours from younger patients displayed higher CIN70 scores but were unexpectedly enriched within the GS group derived by the 12-gene genomic instability signature. Taken together, these data suggest that CIN may be a particular feature of younger-onset ER negative breast cancers, that may define outcome in this high-risk group.

These data are somewhat surprising given the potential for aneuploidy and genome instability to be generated in aging somatic cells [14-16]. Several lines of evidence support the propensity for somatic cells to generate aneuploidy and mitotic errors with age. Specific changes in gene expression associated with the kinetochore, centromere, microtubule and spindle assembly apparatus have been associated with age-dependent aneuploidy [14, 15]. In addition, continued telomeric attrition and senescence failure may generate mitotically unstable chromosomes through breakage-fusion-bridge cycles [17, 18]. Therefore, from these data, it might be predicted that aneuploidy may be enriched in tumours from older patients. In contrast, we find that total functional aneuploidy, measured by the CIN70 expression signature[13], appears to be enriched in tumours from younger patients with ER negative breast cancer. These data suggest the need to identify an underlying mechanistic basis for this association of younger age at diagnosis with CIN in ER negative breast cancer.

Gene expression changes have previously been identified that characterise breast tumours in younger patients. Anders and colleagues investigated the age-specific differences in prognosis, clinicopathologic variables and gene expression patterns between younger (45 years) and older (65 years) women with breast cancer and found a distinct set of genes associated with the younger cohort – suggesting there might exist age-related differences at the molecular level [19]. However, it is difficult to draw conclusions as to whether these differences represent varying prevalence of distinct subtypes and tumour grade between distinct age-cohorts or whether they reflect other age related differences [20]. More recent work by Anders has suggested that the gene expression patterns uniting the younger cohort may relate to an enrichment of basal-like tumours in this patient group [21]. Our data, using two independently derived expression measures of CIN, suggest that a molecular feature of tumours from younger patients with ER negative breast cancers may reflect structural or numerical CIN. Consistent with an association of CIN with higher risk features, we show here in our meta-analysis that ER negative tumours are enriched for CIN, supporting our previous analysis in a smaller cohort [11]. These data are in agreement with data from Habermann and colleagues suggesting that genome instability is a unifying feature of higher risk tumours [12] and tumours from younger patients display higher expression of surrogate of measurements of tumour growth and genomic instability [22].

These data should be interpreted with caution and require validation by more direct measurements of CIN, such as centromeric FISH, rather than our surrogate measure using the CIN70 expression signature. Notably, we observe concordance for the association of younger age with CIN using both CIN expression measures in ER negative but not ER positive breast cancers. The reasons for this discordance remain unclear, however, others have also failed to demonstrate increased structural chromosomal complexity in ER positive tumours from younger patients by CGH [23].

However, evidence that there may be a propensity for increased chromosomal instability in breast cancers from younger patients with ER negative breast cancer suggests the need for such validation approaches and a requirement for a greater understanding of the molecular drivers of structural and numerical chromosomal instability in ER negative breast cancer that may account for this age-related disparity.

## METHODS

### Microarray Expression Data

Publicly available expression microarray data were obtained for 9 breast cancer datasets[24-30] and “GSE2109”, including 1772 unique patients. Additionally, we used four publicly available neo-adjuvant datasets representing 651 unique patients [31-34].

### Data Analysis

For datasets measured on Affymetrix HG-U133A, HT-HG-U133A or HG-U133 Plus 2.0 platforms, we estimated ER and HER2 status by k-medoids clustering of the affymetrix probe sets corresponding to the ESR1 (205225_at) and ERBB2 (216836_s_at) genes according to published methods [35]. We estimated CIN70 scores by computing the mean expression of the probe sets matching the 70 genes comprising the CIN70 signature as described [11, 13]. For microarray platforms where not all 70 genes were present on the array platform, we computed the mean over all CIN70 genes present on the particular platform. For all analyses, CIN70 scores were first estimated for each dataset separately and normalized to a standard normal distribution. The normalized CIN70 scores of all datasets were then combined and CIN70 score quartiles were defined based on the combined CIN70 scores. Additionally, all patients were grouped into genomically unstable (GU) and genomically stable (GS) patients based on a 12 gene genomic instability signature derived by Habermann et al[12]. The expression of all genes matching the 12 gene signature was utilised to cluster the data into GS and GU patients by k-medoids clustering. For further analysis, we used the average expression values of genes matching several probe sets. The cluster membership was derived by comparing the expression direction of the genes in the signature with published results [12]. The To assess the association of the GS and GU groups or CIN70 scores with age, grade, size, lymph node, HER2 and ER status all datasets, where the particular information was available, were combined.

### Statistical Analysis

All breast cancer patients where information about age was available were grouped into young (age < 45) and old (age ≥ 45). Tumours were classified into small (0 – 2 cm) and large (> 2 cm). To assess the difference in CIN70 scores between young and old patients in a meta-nalaysis, a logistic regression model was used to obtain odds ratios for each dataset separately. Additionally, we tested for differences between young and old patients in ER negative and ER positive separately with two-sided t-tests. Cochrane-Armitage trend tests were performed to test for an association of increasing CIN70 quartiles with age, pathological complete response, size, lymph node, HER2 and ER status and to test for a trend of grade 3 versus grade 1 and 2 tumours [36]. We used Fishers exact tests for the comparison of proportions in 2x2 contingency tables. All data analysis and statistics was performed in the R statistical environment version 2.11.1.

## References

[1] Maggard MA, O’Connell JB, Lane KE, Liu JH, Etzioni DA, Ko CY (2003). Do young breast cancer patients have worse outcomes?. The Journal of surgical research.

[2] El Saghir NS, Seoud M, Khalil MK, Charafeddine M, Salem ZK, Geara FB, Shamseddine AI (2006). Effects of young age at presentation on survival in breast cancer. BMC cancer.

[3] Jayasinghe UW, Taylor R, Boyages J (2005). Is age at diagnosis an independent prognostic factor for survival following breast cancer?. ANZ J Surg.

[4] Gabriel CA, Domchek SM (2010). Breast cancer in young women. Breast cancer research : BCR.

[5] Walther A, Houlston R, Tomlinson I (2008). Association between chromosomal instability and prognosis in colorectal cancer: a meta-analysis. Gut.

[6] Sotillo R, Schvartzman JM, Socci ND, Benezra R (2010). Mad2-induced chromosome instability leads to lung tumour relapse after oncogene withdrawal. Nature.

[7] Janssen A, Kops GJ, Medema RH (2009). Elevating the frequency of chromosome mis-segregation as a strategy to kill tumor cells. Proc Natl Acad Sci U S A.

[8] Lee A, Endesfelder D, Rowan A, Walther A, Birkbak N, Futreal AP, Downward J, Szallasi Z, Tomlinson I, Kschischo M, Swanton C (2011). Chromosomal Instability Confers Intrinsic Multi-Drug Resistance. Cancer Res.

[9] Weaver BA, Silk AD, Montagna C, Verdier-Pinard P, Cleveland DW (2007). Aneuploidy acts both oncogenically and as a tumor suppressor. Cancer Cell.

[10] Cahill DP, Kinzler KW, Vogelstein B, Lengauer C (1999). Genetic instability and darwinian selection in tumours. Trends in cell biology.

[11] Birkbak NJ, Eklund AC, Li Q, McClelland SE, Endesfelder D, Tan P, Tan IB, Richardson AL, Szallasi Z, Swanton C (2011). Paradoxical relationship between chromosomal instability and survival outcome in cancer. Cancer Res.

[12] Habermann JK, Doering J, Hautaniemi S, Roblick UJ, Bundgen NK, Nicorici D, Kronenwett U, Rathnagiriswaran S, Mettu RK, Ma Y, Kruger S, Bruch HP, Auer G, Guo NL, Ried T (2009). The gene expression signature of genomic instability in breast cancer is an independent predictor of clinical outcome. Int J Cancer.

[13] Carter SL, Eklund AC, Kohane IS, Harris LN, Szallasi Z (2006). A signature of chromosomal instability inferred from gene expression profiles predicts clinical outcome in multiple human cancers. Nat Genet.

[14] Geigl JB, Langer S, Barwisch S, Pfleghaar K, Lederer G, Speicher MR (2004). Analysis of gene expression patterns and chromosomal changes associated with aging. Cancer research.

[15] Ly DH, Lockhart DJ, Lerner RA, Schultz PG (2000). Mitotic misregulation and human aging. Science.

[16] Richardson B (2003). Impact of aging on DNA methylation. Ageing research reviews.

[17] Hackett JA, Feldser DM, Greider CW (2001). Telomere dysfunction increases mutation rate and genomic instability. Cell.

[18] Artandi SE, Chang S, Lee SL, Alson S, Gottlieb GJ, Chin L, DePinho RA (2000). Telomere dysfunction promotes non-reciprocal translocations and epithelial cancers in mice. Nature.

[19] Anders CK, Acharya CR, Hsu DS, Broadwater G, Garman K, Foekens JA, Zhang Y, Wang Y, Marcom K, Marks JR, Mukherjee S, Nevins JR, Blackwell KL, Potti A (2008). Age-specific differences in oncogenic pathway deregulation seen in human breast tumors. PloS one.

[20] Thomas GA, Leonard RC (2009). How age affects the biology of breast cancer. Clinical oncology.

[21] Anders CK, Fan C, Parker JS, Carey LA, Blackwell KL, Klauber-DeMore N, Perou CM Breast carcinomas arising at a young age: unique biology or a surrogate for aggressive intrinsic subtypes? Journal of clinical oncology : official journal of the American Society of Clinical Oncology. 2011.

[22] Eppenberger-Castori S, Moore DH, Thor AD, Edgerton SM, Kueng W, Eppenberger U, Benz CC (2002). Age-associated biomarker profiles of human breast cancer. The international journal of biochemistry & cell biology.

[23] Yau C, Fedele V, Roydasgupta R, Fridlyand J, Hubbard A, Gray JW, Chew K, Dairkee SH, Moore DH, Schittulli F, Tommasi S, Paradiso A, Albertson DG, Benz CC (2007). Aging impacts transcriptomes but not genomes of hormone-dependent breast cancers. Breast cancer research : BCR.

[24] Chin K, DeVries S, Fridlyand J, Spellman PT, Roydasgupta R, Kuo WL, Lapuk A, Neve RM, Qian Z, Ryder T, Chen F, Feiler H, Tokuyasu T, Kingsley C, Dairkee S, Meng Z (2006). Genomic and transcriptional aberrations linked to breast cancer pathophysiologies. Cancer Cell.

[25] Miller LD, Smeds J, George J, Vega VB, Vergara L, Ploner A, Pawitan Y, Hall P, Klaar S, Liu ET, Bergh J (2005). An expression signature for p53 status in human breast cancer predicts mutation status, transcriptional effects, and patient survival. Proc Natl Acad Sci U S A.

[26] Wang Y, Klijn JG, Zhang Y, Sieuwerts AM, Look MP, Yang F, Talantov D, Timmermans M, Meijer-van Gelder ME, Yu J, Jatkoe T, Berns EM, Atkins D, Foekens JA (2005). Gene-expression profiles to predict distant metastasis of lymph-node-negative primary breast cancer. Lancet.

[27] Sotiriou C, Wirapati P, Loi S, Harris A, Fox S, Smeds J, Nordgren H, Farmer P, Praz V, Haibe-Kains B, Desmedt C, Larsimont D, Cardoso F, Peterse H, Nuyten D, Buyse M (2006). Gene expression profiling in breast cancer: understanding the molecular basis of histologic grade to improve prognosis. J Natl Cancer Inst.

[28] Pawitan Y, Bjohle J, Amler L, Borg AL, Egyhazi S, Hall P, Han X, Holmberg L, Huang F, Klaar S, Liu ET, Miller L, Nordgren H, Ploner A, Sandelin K, Shaw PM (2005). Gene expression profiling spares early breast cancer patients from adjuvant therapy: derived and validated in two population-based cohorts. Breast Cancer Res.

[29] Desmedt C, Haibe-Kains B, Wirapati P, Buyse M, Larsimont D, Bontempi G, Delorenzi M, Piccart M, Sotiriou C (2008). Biological processes associated with breast cancer clinical outcome depend on the molecular subtypes. Clin Cancer Res.

[30] Lu X, Wang ZC, Iglehart JD, Zhang X, Richardson AL (2008). Predicting features of breast cancer with gene expression patterns. Breast Cancer Res Treat.

[31] Popovici V, Chen W, Gallas BG, Hatzis C, Shi W, Samuelson FW, Nikolsky Y, Tsyganova M, Ishkin A, Nikolskaya T, Hess KR, Valero V, Booser D, Delorenzi M, Hortobagyi GN, Shi L (2010). Effect of training-sample size and classification difficulty on the accuracy of genomic predictors. Breast cancer research : BCR.

[32] Farmer P, Bonnefoi H, Anderle P, Cameron D, Wirapati P, Becette V, Andre S, Piccart M, Campone M, Brain E, Macgrogan G, Petit T, Jassem J, Bibeau F, Blot E, Bogaerts J (2009). A stroma-related gene signature predicts resistance to neoadjuvant chemotherapy in breast cancer. Nat Med.

[33] Hess KR, Anderson K, Symmans WF, Valero V, Ibrahim N, Mejia JA, Booser D, Theriault RL, Buzdar AU, Dempsey PJ, Rouzier R, Sneige N, Ross JS, Vidaurre T, Gomez HL, Hortobagyi GN (2006). Pharmacogenomic predictor of sensitivity to preoperative chemotherapy with paclitaxel and fluorouracil, doxorubicin, and cyclophosphamide in breast cancer. J Clin Oncol.

[34] Bonnefoi H, Potti A, Delorenzi M, Mauriac L, Campone M, Tubiana-Hulin M, Petit T, Rouanet P, Jassem J, Blot E, Becette V, Farmer P, Andre S, Acharya CR, Mukherjee S, Cameron D (2007). Validation of gene signatures that predict the response of breast cancer to neoadjuvant chemotherapy: a substudy of the EORTC 10994/BIG 00-01 clinical trial. The lancet oncology.

[35] Gong Y, Yan K, Lin F, Anderson K, Sotiriou C, Andre F, Holmes FA, Valero V, Booser D, Pippen JE, Vukelja S, Gomez H, Mejia J, Barajas LJ, Hess KR, Sneige N (2007). Determination of oestrogen-receptor status and ERBB2 status of breast carcinoma: a gene-expression profiling study. The lancet oncology.

[36] Armitage P (1955). Tests for Linear Trends in Proportions and Frequencies. Biometrics.

